# Supplementary material for: Features influencing the health and economic impact of preventing COVID-19 in immunocompromised individuals
Source: PLoS Comput Biol. 2025 Jun 2;21(6):e1013072. doi: 10.1371/journal.pcbi.1013072 (PMC12129335; doi:10.1371/journal.pcbi.1013072)
Supplement: S1 Text. Supplementary Information — Fig A. Diagram of the model and the relationships between variables and parameters. Fig B. Diagram showing a probability tree for COVID-19 disease progression. Fig C. A mathematical relationship between utility and the degree of shielding. Fig D. (a) Incremental cost-effectiveness plane and (b) a cost-effectiveness acceptability curve with a time horizon of 5 years when the shielding cost is considered. Fig E. Cost-effectiveness acceptability curves when the cost of shielding is included. Fig F. One-way sensitivity analysis between incremental costs, incremental QALYs and ICER and features influencing the health and economic impact of the LAAB-PrEP over different time-horizons of 2 years (a,d, g), 5 years (b, e, h) and 10 years (c, f, i) when the shielding cost is considered. Fig G. Correlations between incremental QALYs and features influencing the health impact of the LAAB-PrEP and shielding combination based on different time-horizons of 2 years (a), 5 years (b) and 10 years (c). Table A. Definitions of the variables used in the model structure in Figs A and B in S1 Text. Table B. Definitions of the parameters used for the calculations and, where relevant, the distributions used for the probabilistic sensitivity analysis. Table C. Summary of parameters in risk subgroups of immunocompromised individuals, as defined by the INFORM study. Table D. Definitions of parameters used to calculate the upper bound of the weekly cost of shielding. (DOCX) [file pcbi.1013072.s001.docx]

**Supporting Information**

**Features influencing the health and economic impact of preventing COVID-19 in immunocompromised individuals**

**Short title:** Long-acting antibody prophylaxis against COVID-19

**Authors:** Siyu Chen^1^, Sudhir Venkatesan^2^, Sofie Arnetorp^3^, Klas Bergenheim^3^, Sabada Dube^4^, Wilhelmine Meeraus^4^, Cátia Ferreira^5^, Sylvia Taylor^6^, Lisa J White^7,8^*

**Affiliations:**

^1^Department of Public and Ecosystem Health, College of Veterinary Medicine, Cornell University, Ithaca, New York, USA

^2^Medical and Payer Evidence, BioPharmaceuticals Medical, AstraZeneca, Cambridge, UK

^3^Health Economics & Payer Evidence, BioPharmaceuticals R&D, AstraZeneca, Gothenburg, Sweden

^4^Medical Evidence, Vaccines and Immune Therapies Unit, AstraZeneca, Cambridge, UK

^5^Vaccines and Immune Therapies, BioPharmaceuticals Medical, AstraZeneca, Wilmington, Delaware, USA

^6^Self-employed, Cambridge, UK

^7^Model Health Ltd, Oxford, UK

^8^Department of Biology, University of Oxford, UK

* lisa.white@modelhealth.online; lisa.white@biology.ox.ac.uk

A coupled mechanistic and economic model

The model allows a description of the baseline standard of care (shielding), representing the comparator, and the designing of an alternative scenario, with various combinations of a new intervention, LAAB-PrEP, with the existing one, i.e., shielding (Fig 5 and S1 Fig). If $i=0$ denotes the comparator group (shielding) and $i=1$ denotes the intervention group (a combination of LAAB-PrEP and shielding), then the full equations are given by Equation 1.

$$\frac{dS_{i}(t)}{dt}=-\left( 1-\epsilon_{Ci} \right)\lambda_{i}S_{i}-\mu S_{i}+\upsilon_{C}I_{Ci}+\left( 1-\delta_{H} \right)\upsilon_{H}I_{Hi}+\left( 1-\delta_{U} \right)\upsilon_{U}I_{Ui}+\left( 1-\delta_{V} \right)\upsilon_{V}I_{Vi}$$

$$\frac{dI_{Ci}(t)}{dt}=\left( 1-\epsilon_{Ci} \right)\left( 1-{\left( 1-\epsilon_{Hi} \right)p}_{H} \right){\lambda_{i}S}_{i}-{(\upsilon}_{C}{+\mu)I}_{Ci}$$

$$\frac{dI_{Hi}(t)}{dt}=\left( 1-\epsilon_{Ci} \right)\left( 1-\epsilon_{Hi} \right)p_{H}\left( 1-p_{U} \right){\lambda_{i}S}_{i}-(\upsilon_{H}+\mu)I_{Hi}$$

$$\frac{dI_{Ui}(t)}{dt}=\left( 1-\epsilon_{Ci} \right)\left( 1-\epsilon_{Hi} \right)p_{H}p_{U}\left( 1-p_{V} \right)\lambda_{i}S_{i}-{(\upsilon}_{U}+\mu)I_{Ui}$$

$$\frac{dI_{Vi}(t)}{dt}=\left( 1-\epsilon_{Ci} \right)\left( 1-\epsilon_{Hi} \right){p_{H}p_{U}p}_{V}\lambda_{i}S_{i}-(\upsilon_{V}+\mu)I_{Vi}$$

$$\frac{dM_{i}(t)}{dt}=\mu{(S}_{i}+I_{Ci}+I_{Hi}+I_{Ui}+I_{Vi})+\delta_{C}{\upsilon_{C}I}_{Ci}+\delta_{H}\upsilon_{H}I_{Hi}+\delta_{U}\upsilon_{U}I_{Ui}+\delta_{V}\upsilon_{V}I_{Vi}$$

$$\frac{dC_{Hi}\left( t \right)}{dt}=\left( 1-k_{c} \right)^{t}\left[ c_{H}\left( 1-\epsilon_{Ci} \right)\left( 1-\epsilon_{Hi} \right)p_{H}\left( 1-p_{U} \right){\lambda_{i}S}_{i}\left( L_{MH}\delta_{H}+L_{MH}\left( 1-\delta_{H} \right) \right)+c_{U}\left( 1-\epsilon_{Ci} \right)\left( 1-\epsilon_{Hi} \right)p_{H}p_{U}\left( 1-p_{V} \right)\lambda_{i}S_{i}\left( L_{MU}\delta_{U}+L_{MU}\left( 1-\delta_{U} \right) \right)+c_{V}\left( 1-\epsilon_{Ci} \right)\left( 1-\epsilon_{Hi} \right){p_{H}p_{U}p}_{V}\lambda_{i}S_{i}\left( L_{MV}\delta_{V}+L_{MV}\left( 1-\delta_{V} \right) \right) \right]$$

$$\upsilon_{H}=\frac{1-\delta_{H}}{L_{NH}}+\frac{\delta_{H}}{L_{MH}}$$

$$\upsilon_{U}=\frac{1-\delta_{U}}{L_{NU}}+\frac{\delta_{U}}{L_{MU}}$$

$$\upsilon_{V}=\frac{1-\delta_{V}}{L_{NV}}+\frac{\delta_{V}}{L_{MV}}$$

(Equation 1)

Equation 1 was numerically solved using R software (version 4.1.2) with the following initial conditions:

$$S_{i}\left( t=0 \right)=Y$$

$$I_{Ci}\left( t=0 \right)=1$$

$$I_{Hi}\left( t=0 \right)=0$$

$$I_{Ui}\left( t=0 \right)=0$$

$$I_{Vi}\left( t=0 \right)=0$$

$$M_{i}\left( t=0 \right)=0$$

$$C_{Hi}\left( t=0 \right)=0$$

(Equation 2)

where $Y$ is the total population size, assumed to be 1,000 for this model.

We assumed that the dosing interval for patients is equal to the duration of protection of a dose of LAAB-PrEP ($l$ years). We then aggregated the dosing costs for LAAB-PrEP every $l$ years. The discounted cost of LAAB-PrEP rollout, $C_{d}$, is given by Equation 3.

$$C_{d}=c_{d}\sum_{j=0}^{\left\lfloor T/l \right\rfloor} \left[ {(1-k_{c})}^{\Delta_{2}j}(S_{1}(\Delta_{2}j)+I_{C1}(\Delta_{2}j)+I_{H1}(\Delta_{2}j)+I_{U1}(\Delta_{2}j)+I_{V1}(\Delta_{2}j)) \right]$$

(Equation 3)

$$\Delta_{2}=\frac{l}{\Delta_{1}}$$

(Equation 4)

Here, $\Delta_{1}(\Delta_{1}<1)$ denotes the output time step of the solution of the differential equations defined in Equation 1. $\left\lfloor x \right\rfloor$ is a floor function and outputs the greatest integer less than or equal to $x$. In our analysis, $l$ is fixed at 6 months.

Considering the cost of shielding, $c_{s}$, is parameterised as per week, we aggregated the total discounted cost of shielding (furlough) per week with LAAB-PrEP and without LAAB-PrEP by $C_{s1}$ and $C_{s0}$, respectively.

$$C_{s1}=c_{s}\sum_{j=0}^{\left\lfloor T/l \right\rfloor} \left[ {(1-k_{c})}^{\Delta_{3}j}(S_{1}(\Delta_{3}j)+I_{C1}(\Delta_{3}j)+I_{H1}(\Delta_{3}j)+I_{U1}(\Delta_{3}j)+I_{V1}(\Delta_{3}j)) \right]$$

(Equation 5)

$$C_{s0}=c_{s}\sum_{j=0}^{\left\lfloor T/l \right\rfloor} \left[ {(1-k_{c})}^{\Delta_{3}j}(S_{0}(\Delta_{3}j)+I_{C0}(\Delta_{3}j)+I_{H0}(\Delta_{3}j)+I_{U0}(\Delta_{3}j)+I_{V0}(\Delta_{3}j)) \right]$$

(Equation 6)

$$\Delta_{3}=\frac{1}{{52\Delta}_{1}}$$

(Equation 7)

The life-years in the comparator and intervention groups can be calculated by Equations 8 and 9, respectively:

$$LY_{0}=\int_{0}^{T} \left( Y-M_{0}\left( t \right) \right)\left( 1-k_{y} \right)^{t}dt$$

(Equation 8)

$$LY_{1}=\int_{0}^{T} \left( Y-M_{1}\left( t \right) \right)\left( 1-k_{y} \right)^{t}dt$$

(Equation 9)

The quality-adjusted life-years (QALYs) in the comparator and intervention groups are the product of life-years calculated using Equations 8 and 9 and the discounting factor:

$$QALY_{0}=\left( u_{0}\left( 1-\left( 1-u_{s} \right)\left( 1-\eta\right) \right) \right)LY_{0}$$

(Equation 10)

$$QALY_{1}=\left( u_{0}\left( 1-\left( 1-u_{s} \right)\left( 1-\eta\right)\left( 1-\rho\right) \right) \right){LY}_{1}$$

(Equation 11)

By discretising the integration (Equations 10 and 11) using Euler’s method with output time step $\Delta_{1}(\Delta_{1}<1)$, we obtain:

$$QALY_{0}=\Delta_{1}\left( u_{0}\left( 1-\left( 1-u_{s} \right)\left( 1-\eta\right) \right) \right)\sum_{j=1}^{T/\Delta_{1}} \left( Y-M_{0}\left( j \right) \right)\left( 1-k_{y} \right)^{\Delta_{1}j}$$

(Equation 12)

$$QALY_{1}=\Delta_{1}(u_{0}(1-(1-u_{s})(1-\eta)(1-\rho)))\sum_{j=1}^{T/\Delta_{1}} \left( Y-M_{1}\left( j \right) \right)\left( 1-k_{y} \right)^{\Delta_{1}j}$$

(Equation 13)

The upper bound of shielding (furlough) cost is approximated by

$$max(w\times r\times p)$$

(Equation 14)

where $w$ is the average weekly salary in England, $r$ is the furlough payment rate made by the government, and $p$ is the percentage of immunocompromised individuals in England who were on furlough in 2020. We approximated $p$ as the proportion of individuals in England who were shielding and who were on furlough, based on the English Office for National Statistics Shielding Behavioural Survey [1]. The ranges of $w, r$and $p$ are listed in S4 Table.

Table A. Definitions of the variables used in the model structure in S1 and S2 Figs, if $i=0$ denotes the comparator group (shielding) and $i=1$ denotes the intervention group (a combination of LAAB-PrEP and shielding).

| Symbol | Definition |
| --- | --- |
| $S_{i}$ | Susceptible |
| $I_{Ci}$ | Clinically insignificant disease |
| $I_{Hi}$ | Severe disease: general hospital bed |
| $I_{Ui}$ | Severe disease: ICU bed and not on an invasive ventilator |
| $I_{Vi}$ | Severe disease: ICU bed and on an invasive ventilator |
| $C_{Hi}$ | Cumulative cost for hospital treatment |
| $M_{i}$ | Cumulative mortality for any reasons |

Table B. Definitions of the parameters used for the calculations and, where relevant, the distributions used for the probabilistic sensitivity analysis.

| Category | Symbol | Definition | Distribution of parameters | Unit | Reference |
| --- | --- | --- | --- | --- | --- |
| Epidemiology | $y_{H}$ | Incidence risk of hospitalisation due to COVID-19 infection | Uniform (0.005, 0.05) | Per person per year | [2] |
|  | $\upsilon_{C}$ | 1 / duration of symptomatic infection | gamma (7, 1) | Day^-1^ | [3-5] |
|  | $L_{MH}$ | Length of stay in a general hospital bed (resulting in death) | Uniform (5.79, 7.28) | Day | [6] |
|  | $L_{MU}$ | Length of stay in ICU and not on a ventilator (resulting in death) | Uniform (7.7, 24.7) | Day | [6] |
|  | $L_{MV}$ | Length of stay in ICU and on a ventilator (resulting in death) | Uniform (18.8, 41.5) | Day | [6] |
|  | $L_{NH}$ | Length of stay in a standard hospital bed (and discharged alive) | Uniform (5.79, 7.28) | Day | [6] |
|  | $L_{NU}$ | Length of stay in ICU and not on a ventilator (and discharged alive) | Uniform (7.7, 24.7) | Day | [6] |
|  | $L_{NV}$ | Length of stay in ICU and on a ventilator (and discharged alive) | Uniform (18.8, 41.5) | Day | [6] |
| Shielding | $\sigma$ | Shielding effectiveness in the absence of LAAB-PrEP | Uniform (0, 1) | / | / |
|  | $\rho$ | Reduction in shielding due to protection with LAAB-PrEP | Uniform (0, 1) | / | / |
|  | $c_{s}$ | Shielding cost per week | Uniform (99.35, 196.8) | £ | [7-9]; see also S4 Table; only used in the further sensitivity analysis for S4-S7 Figs. |
|  | $u_{s}$ | Quality of life adjusted for shielding | Uniform (0.75,1) | / | [10, 11] |
| Risk group | $\mu$ | 1 / (average time to death in a risk subgroup in the absence of COVID-19) | Uniform (1/50, 1/5) | Year^-1^ | [2] |
|  | $\delta_{H}$ | Probability of dying if in a general hospital bed | Uniform (0.1, 0.5) | / | [2] |
|  | $\delta_{U}$ | Probability of dying if in ICU and not on a ventilator | Uniform (0.1, 0.5) | / | [2] |
|  | $\delta_{V}$ | Probability of dying if in ICU and on a ventilator | Uniform (0.1, 0.5) | / | [2] |
|  | $p_{H}$ | Probability of requiring general hospital bed  treatment given symptoms | Uniform (0.02, 0.1) | / | [2] |
|  | $p_{U}$ | Probability of requiring ICU without ventilator treatment given hospitalisation | Uniform (0.001, 0.1) | / | [2] |
|  | $p_{V}$ | Probability of requiring ventilator treatment after admission to ICU | Uniform (0.001, 0.1) | / | [2] |
|  | $u_{0}$ | Quality of life for risk group in absence of COVID-19 infection | Uniform (0.75,0.85) | / |  |
| LAAB-PrEP effectiveness | $\epsilon_{C}$ | Effectiveness of prophylaxis against symptomatic disease (endpoint) | Uniform (0.1, 0.9) | / | [12] |
|  | $\epsilon_{H}$ | Effectiveness of prophylaxis  against hospitalisation (endpoint) | Uniform (0, 0.5) | / | [12] |
| Economics | $c_{d}$ | Cost per dose of LAAB-PrEP | Uniform (500, 2500) | £ | [13] |
|  | $c_{H}$ | Cost per day in a general hospital bed | Uniform (372.81, 677.02) | £ | [14] |
|  | $c_{U}$ | Cost per day in ICU | Uniform (603.93, 1333.58) | £ | [14] |
|  | $c_{V}$ | Cost per day in ICU and on a ventilator | Uniform (982.35, 2167.58) | £ | [14] |
|  | $T$ | Time horizon | 2 or 5 or 10 | Year | / |
|  | $k_{c}$ | Discounting rate of cost | 3.5% | / | [15] |
|  | $k_{y}$ | Discounting rate of life-years | 3.5% | / | [15] |

Note: the ranges of parameters $y_{H}$, $p_{H}$, $p_{U}$ and $\mu$ are informed by the INFORM study [2] and are also summarised in Table S3.

Table C. Summary of parameters in risk subgroups of immunocompromised individuals, as defined by the INFORM study; see Tables 2 and 3 in Evans et al. (2023) [2].

| Risk group | Incidence risk of hospitalisation due to COVID-19 infection | Probability of requiring general hospital bed  treatment given symptoms | Probability of requiring ICU without ventilator treatment given hospitalisation | 1 / (average time to death in a risk subgroup in the absence of COVID-19) |
| --- | --- | --- | --- | --- |
|  | $y_{H}$ | $p_{H}$ | $p_{U}$ | $\mu$ |
| Primary immunodeficiency | (0.015, 0.025) | (0.06, 0.08) | (0.001, 0.1) | (0.045, 0.05) |
| Secondary immunodeficiency | (0.009, 0.02) | (0.05,0.07) | (0.001, 0.1) | (0.045, 0.055) |
| High-dose, long-term, moderate dose corticosteroid treatment | (0.007, 0.01) | (0.03, 0.05) | (0.001, 0.002) | (0.07, 0.08) |
| End-stage renal disease or dialysis | (0.02, 0.03) | (0.07, 0.08) | (0.055, 0.065) | (0.08, 0.12) |
| Organ transplant > 5 years and ≤ 7 years prior | (0.02, 0.03) | (0.075, 0.085) | (0.15, 0.25) | (0.025, 0.035) |
| Solid tumour in the past five years | (0.005, 0.015) | (0.03, 0.07) | (0.01, 0.07) | (0.08, 0.2) |
| Haematological malignancy in the past five years | (0.01, 0.05) | (0.06, 0.15) | (0.03, 0.09) | (0.06, 0.15) |
| Advanced or untreated HIV | (0.009, 0.02) | (0.04, 0.08) | (0, 0.05) | (0.05, 0.2) |

Table D. Definitions of parameters used to calculate the upper bound of the weekly cost of shielding.

| Definition | Symbol | Range | Unit | Reference |
| --- | --- | --- | --- | --- |
| Average weekly salary in England | $w$ | [300, 620] | £ | [7] |
| Furlough payment rate | $r$ | [0.6, 0.8] | / | [9] |
| Percentage of immunocompromised individuals in England who were on furlough in 2020 | $p$ | [0.35, 0.41] | / | [8] |

Fig A. Diagram of the model and the relationships between variables and parameters.

Fig B. Diagram showing a probability tree for COVID-19 disease progression.

Fig C. A mathematical relationship between utility and the degree of shielding. The x-axis is the degree of shielding, varying from 0 to 100%, while the y-axis is utility, also varying from 0 to 100%. If an immunocompromised individual does not shield, i.e., 𝑠=0, the utility is $u_{0}$<1, due to their primary condition. If an immunocompromised individual shields 100%, i.e. 𝑠=1, the utility is the product of $u_{0}$ and $u_{s}$, where $u_{s}$ is the discounting impact of shielding on the utility value of an immunocompromised individual’s life.

Further sensitivity analysis


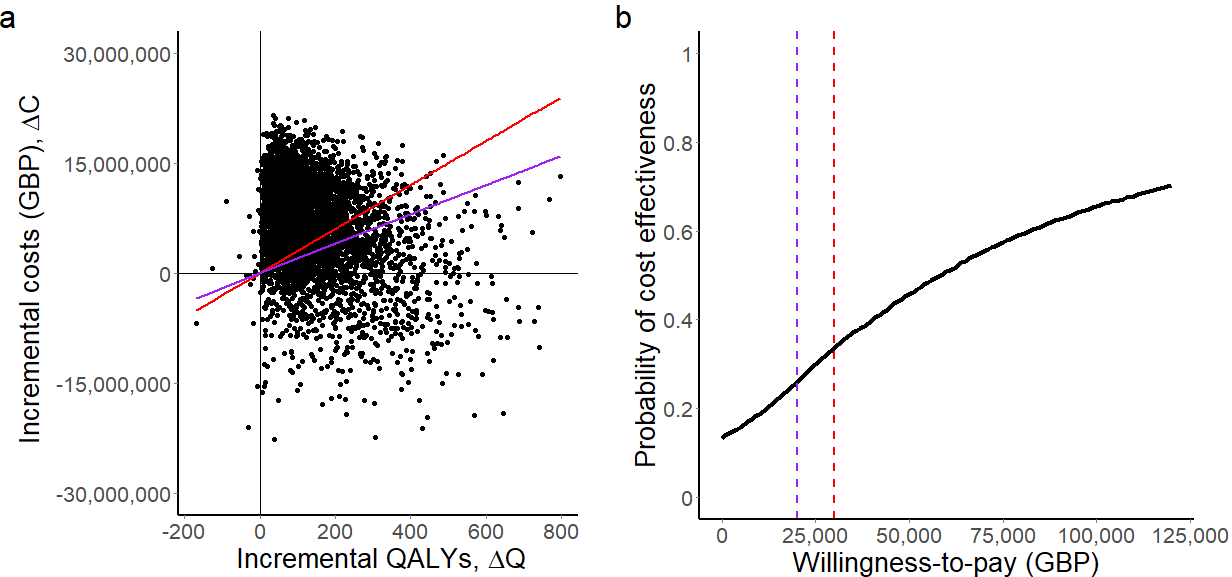


Fig D. (**a**) Incremental cost-effectiveness plane and (**b**) a cost-effectiveness acceptability curve with a time horizon of 5 years when the shielding cost is considered. (**a**) shows the cost-effectiveness planes of costs based on 5,000 bootstrap replications for 1,000 hypothetical patients when shielding cost is considered. The mean incremental QALYs and incremental costs are approximately 140 and 6,300,000 GBP, respectively. The purple and red solid lines are 20,000 and 30,000 GBP per incremental QALY, respectively. (**b**) shows the cost-effectiveness acceptability indicating the probability that the intervention (a mixture of shielding and LAAB-PrEP) is cost effective compared with the standard of care (shielding) for a given willingness-to-pay threshold with shielding costs considered. The y-axis represents the probability that the intervention will be cost effective, and the x-axis represents a range of values for the willingness-to-pay threshold. The purple and red dashed lines represent willingness-to-pay thresholds of 20,000 and 30,000 GBP, respectively. The discounting rate for both costs and QALYs is 3.5%.


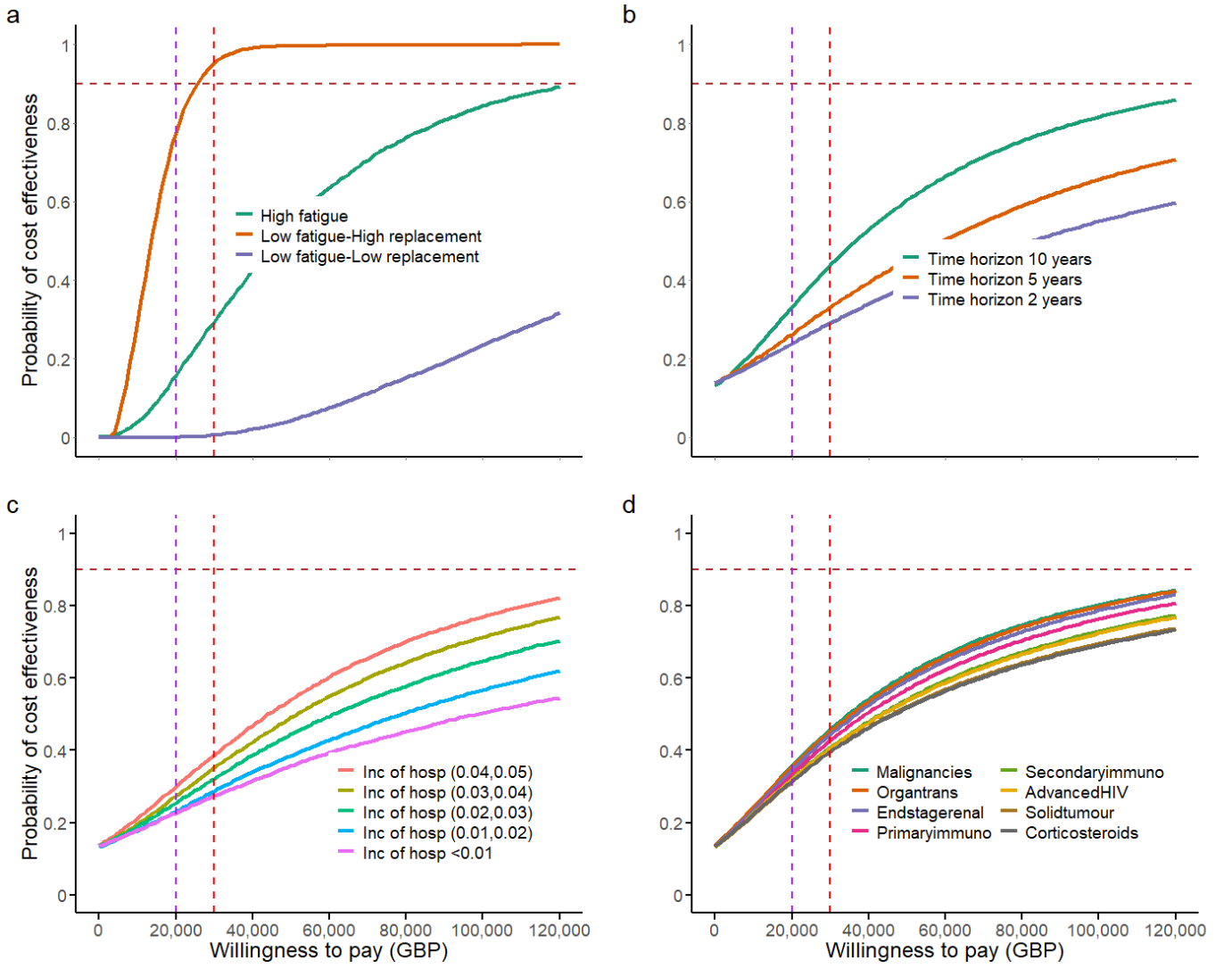


Fig E. Cost-effectiveness acceptability curves when the cost of shielding is included. The vertical purple and red dashed lines show the willingness-to-pay thresholds of 20,000 and 30,000 GBP, respectively. The horizontal brown dashed line shows the probability of cost effectiveness of 0.9. (**a**) Scenario analysis of behavioural parameters. The low fatigue, high replacement scenario is represented by the red line ( $\eta\in\left[ 0, 0.10 \right]$ and $\rho\in\left[ 0.95, 1 \right]$ ). The high fatigue scenario is represented by the green line ( $\eta\in\left[ 0.75, 0.85 \right]$ ). The low fatigue, low replacement scenario is represented by the purple line ( $\eta\in\left[ 0, 0.15 \right]$ and $\rho\in\left[ 0, 0.1 \right]$ ). (**b**) Sensitivity analysis of time horizons. The green, red and purple lines represent 10, 5 and 2 years, respectively. (**c**) Sensitivity analysis of the incidence of hospitalisation after COVID-19 infection for immunocompromised individuals. The red, yellow, green, blue and pink lines represent the intervals of incidence of hospitalisation as (0.04,0.05), (0.03,0.04), (0.02,0.03), (0.01,0.02) and <0.01 per person per year, respectively. (**d**) Sensitivity analysis of risk subgroups. The legends in (**d**) (from left to right) are ordered from the top line to the bottom line. Details of the characteristics and parameters of each group are described in Table S3 and Evans et al. (2023) [2]. The purple and red vertical dashed lines represent willingness-to-pay thresholds of 20,000 and 30,000 GBP, respectively.


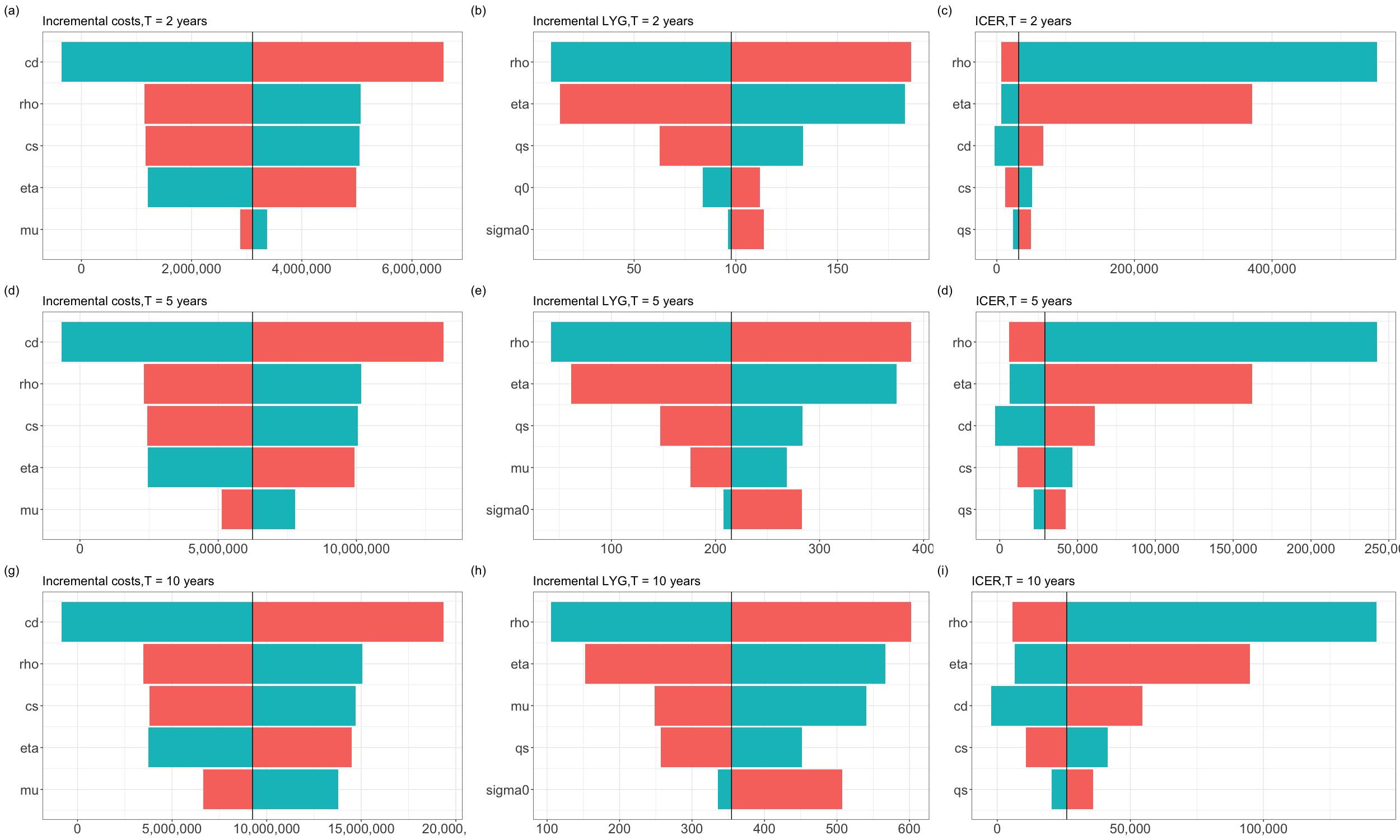


Fig F. One-way sensitivity analysis between incremental costs, incremental QALYs and ICER and features influencing the health and economic impact of the LAAB-PrEP over different time-horizons of 2 years (a, d, g), 5 years (b, e, h) and 10 years (c, f, i) when the shielding cost is considered. The black vertical line in each panel pinpoints the model-estimated values of costs, QALYs and ICER when all the parameters take the mean value in its possible range. Red and blue bars for each labelled parameter indicate the values of costs, QALYs and ICER where the corresponding single parameter took the smallest and largest value in its possible range and all the remaining parameters were sampled from the entire possible range. A blue bar on the right-hand side and a red bar on the left-hand side indicate a positive correlation while a red bar on the right-hand side and a blue bar on the left-hand side indicates negative correlations. The parameters positively correlated with incremental costs/incremental QALYs/ICER mean extra costs /QALYs/ICERs will be gained in the intervention group as the parameters increase, while parameters negatively correlated with incremental QALYs/incremental costs/ICER mean QALYs/costs/ICERs will be lost as the parameters decrease in the intervention group.


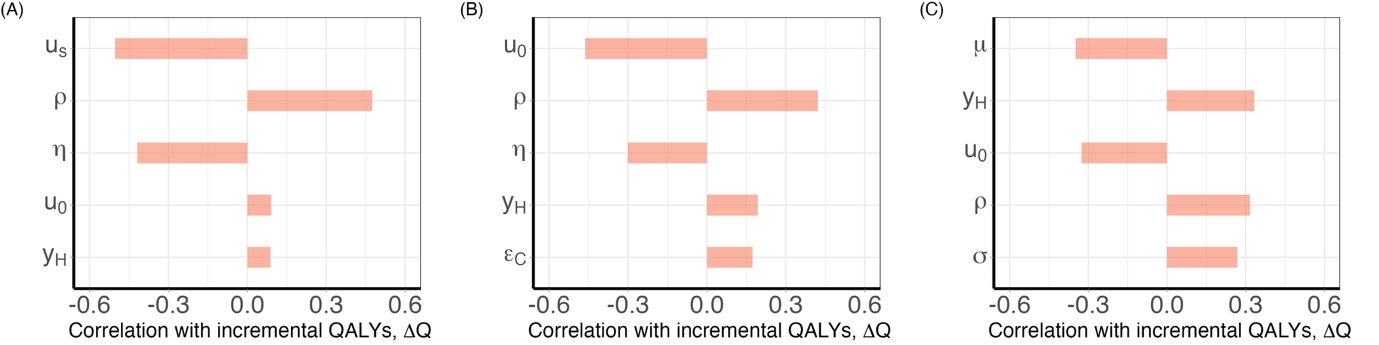


Fig G. Correlations between incremental QALYs and features influencing the health impact of the LAAB-PrEP and shielding combination based on different time-horizons of 2 years (a), 5 years (b) and 10 years (c). Parameters are ranked by the absolute value of their Pearson correlation coefficient with incremental QALYs from top to bottom in each panel. The parameters positively correlated with incremental QALYs mean extra QALYs will be gained in the intervention group as the parameters increase, while parameters negatively correlated with incremental QALYs mean that QALYs will be lost as the parameters decrease in the intervention group.

**References**

1. Office for National Statistics. Coronavirus and shielding of clinically extremely vulnerable people in England: 9 July to 16 July 2020 2020 [17 January 2024]. Available from: <https://www.ons.gov.uk/peoplepopulationandcommunity/healthandsocialcare/conditionsanddiseases/bulletins/coronavirusandshieldingofclinicallyextremelyvulnerablepeopleinengland/latest>.

2. Evans RA, Dube S, Lu Y, Yates M, Arnetorp S, Barnes E, et al. Impact of COVID-19 on immunocompromised populations during the Omicron era: insights from the observational population-based INFORM study. Lancet Reg Health Eur. 2023:100747. doi: <https://doi.org/10.1016/j.lanepe.2023.100747>.

3. Li Q, Guan X, Wu P, Wang X, Zhou L, Tong Y, et al. Early transmission dynamics in Wuhan, China, of novel coronavirus-infected pneumonia. N Engl J Med. 2020;382(13):1199-207. Epub 20200129. doi: 10.1056/NEJMoa2001316. PubMed PMID: 31995857; PubMed Central PMCID: PMCPMC7121484.

4. Davies NG, Kucharski AJ, Eggo RM, Gimma A, Edmunds WJ. Effects of non-pharmaceutical interventions on COVID-19 cases, deaths, and demand for hospital services in the UK: a modelling study. Lancet Public Health. 2020;5(7):e375-e85. Epub 20200602. doi: 10.1016/s2468-2667(20)30133-x. PubMed PMID: 32502389; PubMed Central PMCID: PMCPMC7266572.

5. Byrne AW, McEvoy D, Collins AB, Hunt K, Casey M, Barber A, et al. Inferred duration of infectious period of SARS-CoV-2: rapid scoping review and analysis of available evidence for asymptomatic and symptomatic COVID-19 cases. BMJ Open. 2020;10(8):e039856. Epub 20200805. doi: 10.1136/bmjopen-2020-039856. PubMed PMID: 32759252; PubMed Central PMCID: PMCPMC7409948.

6. Beigel JH, Tomashek KM, Dodd LE, Mehta AK, Zingman BS, Kalil AC, et al. Remdesivir for the treatment of Covid-19 - Final Report. N Engl J Med. 2020;383(19):1813-26. Epub 20201008. doi: 10.1056/NEJMoa2007764. PubMed PMID: 32445440; PubMed Central PMCID: PMCPMC7262788.

7. Office for National Statistics. Average weekly earnings in Great Britain: September 2022 2022 [18 October 2023]. Available from: <https://www.ons.gov.uk/employmentandlabourmarket/peopleinwork/employmentandemployeetypes/bulletins/averageweeklyearningsingreatbritain/september2022>.

8. Office for National Statistics. Coronavirus and shielding of clinically extremely vulnerable people in England, 14 May to 19 May edition of this dataset 2020 [19 October 2023]. Available from: <https://www.ons.gov.uk/peoplepopulationandcommunity/healthandsocialcare/conditionsanddiseases/datasets/coronavirusandshieldingofclinicallyextremelyvulnerablepeopleinengland>.

9. GOV.UK. Calculate how much you can claim using the Coronavirus Job Retention Scheme 2021 [19 October 2023]. Available from: <https://www.gov.uk/guidance/calculate-how-much-you-can-claim-using-the-coronavirus-job-retention-scheme#:~:text=From%201%20July%202021%2C%20the%20government%20will%20pay%2070%25%20of,the%20employee%20is%20on%20furlough>.

10. Di Gessa G, Price D. The impact of shielding during the COVID-19 pandemic on mental health: evidence from the English Longitudinal Study of Ageing. Br J Psych. 2022;221(4):637-43. Epub 2022/04/04. doi: 10.1192/bjp.2022.44.

11. Bachtiger P, Adamson A, Maclean WA, Kelshiker MA, Quint JK, Peters NS. Determinants of shielding behavior during the COVID-19 pandemic and associations with well-being among National Health Service patients: longitudinal observational study. JMIR Public Health Surveill. 2021;7(9):e30460. Epub 20210920. doi: 10.2196/30460. PubMed PMID: 34298499; PubMed Central PMCID: PMCPMC8454693.

12. Levin MJ, Ustianowski A, De Wit S, Launay O, Avila M, Templeton A, et al. Intramuscular AZD7442 (tixagevimab–cilgavimab) for prevention of Covid-19. N Engl J Med. 2022;386(23):2188-200. doi: 10.1056/NEJMoa2116620. PubMed PMID: 35443106.

13. National Institute for Health and Care Excellence. Tixagevimab plus cilgavimab for preventing COVID-19 - technology appraisal guidance TA900 2023 [27 February 2024]. Available from: <https://www.nice.org.uk/guidance/ta900>.

14. Rafia R, Martyn-St James M, Harnan S, Metry A, Hamilton J, Wailoo A. A cost-effectiveness analysis of remdesivir for the treatment of hospitalized patients with COVID-19 in England and Wales. Value Health. 2022;25(5):761-9. Epub 20220220. doi: 10.1016/j.jval.2021.12.015. PubMed PMID: 35197225; PubMed Central PMCID: PMCPMC8858417.

15. National Institute for Health and Care Excellence. NICE health technology evaluations: the manual 2022. Available from: <https://www.nice.org.uk/process/pmg36/resources/nice-health-technology-evaluations-the-manual-pdf-72286779244741>.
