# Supplementary material for: Features influencing the health and economic impact of preventing COVID-19 in immunocompromised individuals
Source: PLoS Comput Biol. 2025 Jun 2;21(6):e1013072. doi: 10.1371/journal.pcbi.1013072 (PMC12129335; doi:10.1371/journal.pcbi.1013072)
Supplement: Attachment — Submitted filename: PCOMPBIOL-D-24-00735_reviewer_REVIEW.pdf [file pcbi.1013072.s003.pdf]

Review of: Features influencing the health and economic impact of long-acting antibody prophylaxis against COVID-19

Thank you for the opportunity to review this manuscript. The authors present an interesting model that looks at the interaction between disutility of shielding and pre-exposure prophylaxis against COVID-19. The paper is clearly written and modelling assumptions clearly explained. However I have some comments on the framing of the paper and some of the assumptions from a health economic perspective.

Specific comments

1. Line 47: “Long-term (5- to 10-year time-horizon) LAAB-PrEP as a repeated dosing prophylaxis strategy against repeated COVID-19 challenges was predicted to be more cost effective than a short-term (6-month time-horizon) strategy.” As the headline concluding sentence in the abstract I think this is potentially misleading. While it is strictly true, based on the analysis presented in the paper neither of these strategies appear likely to be cost-effective using the usual NICE WTP thresholds for England.
2. Line 87: ref (8) does not appear to relate to a definition of shielding.
3. Line 89: Although the UKSA guidance for immunocompromised individuals (ref 9) include suggestions that immunocompromised individuals can take to protect themselves, I am not convinced this constitutes advice to “continue shielding” in the same sense as was recommended to vulnerable individuals during the pandemic.
4. Lines 139 to 138: Although the authors claim that the objective of the study is not to determine whether the LAAB-PrEP is cost-effective due to the large uncertainty in the various model parameters, I think that the study is none-the-less framed as a cost-effectiveness analysis (e.g. displaying a cost-effectiveness plane, and presenting cost-effectiveness acceptability curves as some of the main results), and I think it would therefore be good to accompany the study with a CHEERS reporting checklist for economic evaluations.
5. Line 201: If I have understood the calculation then the “predicted probability” of cost-effectiveness is based upon the proportion of samples shown in Fig 1 A that fall below a given willingness-to-pay threshold, which includes uncertainty in the price and the efficacy of LAAB-PrEP. However, the uniform distributions used for these parameters does not represent the statistical uncertainty in these parameters - rather these parameters are just “not yet known”. It would, therefore, be better to undertake scenario analysis (e.g. a two-way sensitivity analysis) showing how the probability of being cost-effective varies conditional on future LAAB-PrEP products having a particular efficacy and becoming available at a certain price.
6. Line 203, 211, 217 and Fig1: For cost-effectiveness in an English context it would be appropriate to show WTP thresholds of £20000 and £30000 per QALY which are relevant to decisions taken by NICE. I do not think it is useful to show a threshold of £100000. N.B. it should also be noted that for NICE to find something cost-effective using the £30000 per QALY threshold, there would need to be a 90% probability that the true ICER falls below this threshold.
7. Line 206, Fig 1 A: Looking at Fig 1 B, only 30% of simulations are cost-effective at a WTP of 30,000, but in Fig 1 A it appears that the majority of the points fall below the 30,000 WTP line - this is presumably because the number and size of the plotted

points mean many points overlap and no longer visually represent the true density of points.

8. Line 221/Table S3: I could not see the utility values (u0) that are used in the model for the different immunocompromised conditions included in the INFORM study. Another related question is that u0 will presumably vary also with age - is this taken account of in the analysis, and is the modelled population based on the INFORM study representative of the wider population of immunocompromised individuals?
9. Line 257, Fig 2a: Since part of the study objective is to understand how the different parameters influence cost-effectiveness I think it would be useful to show a similar type of figure where the outcome is the ICER rather than QALYs, and rather than showing the level of correlation, show how absolute change in the ICER that results from varying each parameter.
10. Line 266 / Fig 3: I could not see anywhere the specification of which discount rates were used for the scenarios in this figure (or indeed in Fig 1).
11. Line 280: I'm unclear - when the time-horizon is varied from 2 to 5 to 10 years, are the just varying the timeframe over which benefits and costs are counted, or are these also extending the period of time over which an individual is assumed to received LAAB-PrEP?
12. I wasn't clear, does the model include ageing? And if so, does underlying mortality and COVID-19 mortality risk vary with age, which might be important particularly in the in the 10 year time-horizon analysis.
13. Line 355: The authors write that "Typical cost-effectiveness analyses rely on well-defined input values that are used to determine whether a new intervention is cost effective. Our approach differs from this in that it includes uncertain input parameters yet can still be used to determine the features that have the greatest influence on the intervention's potential health impact." I think that the analysis presented is essentially a cost-effective analysis, and the lack of empirical data to inform input parameters is also common to such analysis.
14. Line 367: "We found that shielding behaviour is highly influential in terms of whether LAAB-PrEP is cost-effective" From this it appears that the authors *are* making conclusions about cost-effectiveness. I would further add that I don't think this conclusion is supported by the results - on the contrary from Fig 3 it appears that LAAB-PrEP would not be cost-effective in England using typical NICE thresholds under any of the scenarios presented.
15. Line 375/376: As noted in point 2, I don't think the current guidance represents a strict continuation of the shield guidance that was in place during the pandemic.
16. Line 382: Costs of furlough and SSP are not costs that are born by the NHS, so I do not think they should be included in the base case analysis from a healthcare payer perspective, but could be included in a sensitivity analysis using a societal perspective.
17. Line 401. "However, this does mean that there will be scenarios in which even LAABs with intermediate effectiveness could be cost effective." Again I'm not sure this is supported by the results, as none of the scenarios presented appear likely to be cost-effective.
18. I think the discussion could include a fuller discussion of the study limitations. As one example, it seems very likely that "shielding" behaviour of immunocompromised

individuals could provide protection against a wide range of pathogens beyond SARS-CoV-2, which as far as I can see are not considered in this analysis.

19. Line 443/495: I'm not sure what is meant by the term "model-calibrated". To me this suggests that the model has been in some way fitted to data, but I don't think this is the case. Please could the authors clarify.
20. Line 414: "…we have shown that the expected health **and economic impact** of LAAB-PrEP for COVID-19 in immunocompromised patients will be driven, in order of decreasing importance…" I don't think the analysis has separately shown that the health *and* economic impact are both influenced in this order.
21. Line 512: As noted earlier, I could not see where the discounting sensitivity analysis is presented. Also the reference for the values chosen is the NICE guidelines, but these do not appear to be correct. NICE recommends the use of 3.5% for both costs and QALYs, with 1.5% acceptable in some circumstances that are unlikely to apply to LAAB-PrEP (see page 82 of the guidance document.)
22. Line 520: Here it says for the drug price a gamma distribution was used, but in the appendix it says a uniform distribution was used. As noted earlier, I think this should not be treated as a stochastic parameter, but rather varied in scenario analysis.
