# Supplementary material for: Features influencing the health and economic impact of preventing COVID-19 in immunocompromised individuals
Source: PLoS Comput Biol. 2025 Jun 2;21(6):e1013072. doi: 10.1371/journal.pcbi.1013072 (PMC12129335; doi:10.1371/journal.pcbi.1013072)
Supplement: Attachment — Submitted filename: responses_to_reviewers.docx [file pcbi.1013072.s005.docx]

**Reply to reviewers’ comments**, **submission PCOMPBIOL-D-24-00735**:

"Features influencing the health and economic impact of long-acting antibody prophylaxis against COVID-19"

**Reviewer 1**

***This is a well written paper describing an interesting analysis.***

**Author response:** We appreciate the reviewer’s positive feedback and are very happy to respond to their comments.

1.1. ***AUTHOR SUMMARY. Please clarify whether shielding involves wearing a face mask and only going out when essential or if it is something else. I didn’t understand what shielding meant from this section.***

**Author response:** We thank the reviewer for this observation. We have clarified this in lines 54-57 of the revised manuscript (the clean version without track changes).

1.2. ***INTRODUCTION. This is well written and does a good job of setting the scene for the analysis.***

**Author response:** We appreciate the reviewer’s positive feedback.

1.3. ***RESULTS. Fig 1a – suggest making the dots smaller and/or a lighter colour so you can tell where multiple iterations stack on top of each other. y-axis appears to be wrong (incremental costs of ~$15m)***

**Author response:** We thank the reviewer for this great suggestion. We have revised the dots in Fig 1a with fewer samples and lighter colours. We checked the y-axis, and it was correct: the largest incremental costs are around 15M GBP, but the mean incremental costs are around 6.3M GBP.

1.4. ***Lines 244-247 - I don’t think I follow what this sentence is saying***

**Author response:** We thank the reviewer for pointing out this confusion. We have revised the text in lines 254-263.

1.5. ***Fig 2 – Initially found this strange because the text on 2a and 2c isn’t mentioned until the paragraph beginning at line 280. Is it possible to shift the order around so that this is covered earlier?***

**Author response:** We thank the reviewer for this suggestion and have made the revision as suggested.

1.6. ***Fig 3d is hard to read. Suggest changing colours or making larger.***

**Author response:** We thank the reviewer for this suggestion and have made the revision as suggested.

1.7. ***DISCUSSION. Does the paragraph in lines 373-378 relate at all to the study from South Korea mentioned in lines 316-331?***

**Author response:** We thank the reviewer for this observation. The South Korea study assumed individuals who are immunocompromised experienced similar risks of infection of COVID-19 compared with the general population and did not engage in any shielding behaviour that may reduce their risk of infection. Therefore, the South Korea study is not relevant in the context of our discussion about how shielding behaviour during the peaks of COVID-19 waves would influence the incidence and hospitalisation rates among immunocompromised individuals in the future.

1.8. ***METHODS. Clearly state the perspective for the analysis. Lines 381-382 state that you’re taking the patient and the healthcare sector perspectives—do you mean two different perspectives or the societal perspective?***

**Author response:** We thank the reviewer for this suggestion and have revised lines 421-422 accordingly.

1.9. ***Since this is set in the UK, suggest using a discount rate of 3.5% in the base case to align with NICE recommendations.***

**Author response:** We thank the reviewer for this suggestion. Now we have used a discount rate of 3.5% throughout.

1.10. ***Line 519 – Are you assuming that this drug would only be available in the private sector? What if it became available in the public sector?***

**Author response:** In the UK, the drug is currently only available in the private sector; therefore, all of the cost information was collected through the private sector. We acknowledged that these costs may change if it became available in the public sector, in line 559-562, where we explored a larger parameter space of costs, i.e. 500–2500 GBP (Table S2), in the sensitivity analysis.

1.11. ***Line 526-531 – could you provide further justification for these choices? Is it fair to assume that this is a linear relationship?***

**Author response:** We thank the reviewer for suggesting this improvement. Many social evaluation studies cited in the Introduction, in lines 87-98, have shown that shielding behaviour negatively impacts individuals’ quality of life and well-being. For example, Di Gessa et al. [1] estimated a decreasing linear relationship between shielding patterns and quality of life – those shielding throughout had a lower quality of life ($\beta$= −1.28, 95% CI −2.04 to −0.52) than those who neither shielded nor stayed at home, although prior characteristics and lack of social interactions explain some of this association, even when controlling for all covariates.

We have added an additional reference, which is a longitudinal observational study that investigated the determinants of shielding behaviour during the COVID-19 pandemic and associations with well-being, based on an NHS patient population. They estimated that among individuals who were clinically extremely vulnerable (CEV), shielding was associated with lower ratings of mental well-being (adjusted β −0.40, 95% CI −0.26 to −0.55) and physical well-being (adjusted β −0.51, 95% CI −0.37 to −0.66) [2].

These references demonstrated the relationship, but we expect the values to differ because in our paper we focused on a different population.

1.12. ***Do you include disutility for COVID or just for shielding? Where are the utilities for COVID illness?***

**Author response:** We thank the reviewer for this question. We included disutility for shielding and underlying comorbidities. We omitted the utility of COVID-19 illness because of the relatively shorter time period of infection thus its lower impact on quality of life. We did account for the inflated risk of infection and severe outcomes among immunocompromised individuals after COVID-19 infection.

1.13. ***SUPP. Table S3 – It would be helpful for the column headers to be explained.***

**Author response:** We thank the reviewer for the suggestion. We have added the variable names to the column headers in Table S3.

**Reviewer 2**

***Thank you for the opportunity to review this manuscript. The authors present an interesting model that looks at the interaction between disutility of shielding and pre-exposure prophylaxis against COVID-19. The paper is clearly written, and modelling assumptions clearly explained. However, I have some comments on the framing of the paper and some of the assumptions from a health economic perspective.***

**Author response:** We appreciate the reviewer’s positive feedback and are very happy to respond to their comments.

2.1. ***Line 47: “Long-term (5- to 10-year time-horizon) LAAB-PrEP as a repeated dosing prophylaxis strategy against repeated COVID-19 challenges was predicted to be more cost effective than a short-term (6-month time-horizon) strategy.” As the headline concluding sentence in the abstract, I think this is potentially misleading. While it is strictly true, based on the analysis presented in the paper neither of these strategies appear likely to be cost-effective using the usual NICE WTP thresholds for England.***

**Author response:** To demonstrate the existence of a cost-effective scenario, we changed the parameter ranges and updated Fig 3a. Now it shows that in the newly defined low fatigue and high replacement scenario, the probability of cost effectiveness of the hypothetical LAAB-PrEP is more than 90% given a willingness-to-pay (WTP) threshold of 30,000 GPB and more than 85% given a WTP threshold of 20,000 GBP. We also took on board the reviewer’s suggestion and changed the headline concluding sentence in the Abstract in lines 41-47 of the manuscript.

2.2. ***Line 87: ref (8) does not appear to relate to a definition of shielding.***

**Author response:** We thank the reviewer for reading through the reference. The subheading ‘Shielding’ in Section 5 of reference #8 lays out the guidance about shielding between January and March 2021 for individuals who were CEV: ‘*…Shielding was a voluntary action in which the individual stayed in their home or garden as much as possible, except for leaving their household to attend essential medical appointments or for exercise…’*. We have now explicitly specified this for readers in lines 84-88.

2.3. ***Line 89: Although the UKHSA guidance for immunocompromised individuals (ref 9) include suggestions that immunocompromised individuals can take to protect themselves, I am not convinced this constitutes advice to “continue shielding” in the same sense as was recommended to vulnerable individuals during the pandemic.***

**Author response:** We thank the reviewer for raising this point. The guidance for immunocompromised individuals changed throughout the pandemic, and we have updated the text to reflect this. The issue is whether patients follow the guidance or continue with more stringent or less stringent behavioural protective measures, depending on various factors, including the availability of pharmaceutical alternatives to shielding. The focus of this paper is to explore the influence of these unmeasured choices of future behaviour on the cost-effectiveness of a pharmaceutical option for COVID-19 prophylaxis.

2.4. ***Lines 139 to 138: Although the authors claim that the objective of the study is not to determine whether the LAAB-PrEP is cost-effective due to the large uncertainty in the various model parameters, I think that the study is none-the-less framed as a cost-effectiveness analysis (e.g. displaying a cost-effectiveness plane, and presenting cost-effectiveness acceptability curves as some of the main results), and I think it would therefore be good to accompany the study with a CHEERS reporting checklist for economic evaluations.***

**Author response:** We thank the reviewer for this suggestion. We have added this checklist to the Appendix S4.

2.5. ***Line 201: If I have understood the calculation then the “predicted probability” of cost-effectiveness is based upon the proportion of samples shown in Fig 1 A that fall below a given willingness-to-pay threshold, which includes uncertainty in the price and the efficacy of LAAB-PrEP. However, the uniform distributions used for these parameters does not represent the statistical uncertainty in these parameters –rather these parameters are just “not yet known”. It would, therefore, be better to undertake scenario analysis (e.g. a two-way sensitivity analysis) showing how the probability of being cost-effective varies conditional on future LAAB-PrEP products having a particular efficacy and becoming available at a certain price.***

**Author response:** We have conducted a two-way sensitivity analysis as suggested, shown in Fig S5, and described the findings in lines 263-266. We also conducted a multi-way sensitivity analysis, shown in Fig S8.

2.6. ***Line 203, 211, 217 and Fig1: For cost-effectiveness in an English context, it would be appropriate to show WTP thresholds of £20000 and £30000 per QALY which are relevant to decisions taken by NICE. I do not think it is useful to show a threshold of £100000. N.B. it should also be noted that for NICE to find something cost-effective using the £30000 per QALY threshold, there would need to be a 90% probability that the true ICER falls below this threshold.***

**Author response:** We thank the reviewer for their point about the WTP thresholds. In the NICE guidance, the £20,000–£30,000 per QALY threshold is for the Single Technology Appraisal (STA) process [3,4]. ‘For highly specialised technologies, the NICE committee will consider the size of the incremental QALY gain in relation to the additional weight that would need to be assigned to the QALY benefits for the cost effectiveness of the technology to fall within the highly specialised technologies £100,000 cost per QALY level.’ [3].

We have added purple dashed lines indicating 20,000 GBP per QALY in all relevant figures. We think it is useful to acknowledge that NICE does consider higher WTP thresholds in special cases, i.e., highly specialised technologies in this context, and we have included this as a footnote in lines 224-227. We included brown, horizontal dashed lines to indicate the probability of cost effectiveness of 90% in Fig 3, where the low fatigue and high replacement scenario is >90% likely to be cost effective given the WTP threshold of 30,000 GPB, represented by the red solid lines.

2.7. ***Line 206, Fig 1 A: Looking at Fig 1 B, only 30% of simulations are cost-effective at a WTP of 30,000, but in Fig 1 A it appears that the majority of the points fall below the 30,000 WTP line - this is presumably because the number and size of the plotted points mean many points overlap and no longer visually represent the true density of points***

**Author response:** Please see our responses in 1.3.

2.8. ***Line 221/Table S3: I could not see the utility values (u0) that are used in the model for the different immunocompromised conditions included in the INFORM study. Another related question is that u0 will presumably vary also with age - is this taken account of in the analysis, and is the modelled population based on the INFORM study representative of the wider population of immunocompromised individuals?***

**Author response:** We thank the reviewer for checking this. We did not use any immunocompromised-condition specific utility values (u0) in the model for the different immunocompromised conditions included in the INFORM study because neither the INFORM study nor any existing literature reports u0 for all these immunocompromised conditions. Instead, in the sensitivity analysis, we varied u0 from 0.75 to 0.85 for each individual immunocompromised condition. We also discussed this as a limitation of the study in the Discussion section in lines 394-414.

Ageing is not considered in u0 because for the target population, i.e. immunocompromised individuals, comorbidities play a greater role in disutility than ageing, and ageing in this context is often not linearly correlated with comorbidity.

We did not intend to use the INFORM study to represent the wider population of immunocompromised individuals; instead, we used the INFORM study to show the flexibility of the model framework and as a use case showing how we can parameterise the model for subgroups, where some parameters specific to these groups are scientifically estimated.

2.9. ***Line 257, Fig 2a: Since part of the study objective is to understand how the different parameters influence cost-effectiveness, I think it would be useful to show a similar type of figure where the outcome is the ICER rather than QALYs, and rather than showing the level of correlation, show how absolute change in the ICER that results from varying each parameter.***

**Author response:** We thank the reviewer for this suggestion. We conducted the one-way sensitivity analysis as suggested and visualised the results using tornado plots, shown in Fig 2. We have described the relevant findings in lines 229-244. We have moved the multi-way sensitivity analysis into the supplementary information as Fig S8.

2.10. ***Line 266 / Fig 3: I could not see anywhere the specification of which discount rates were used for the scenarios in this figure (or indeed in Fig 1).***

**Author response:** We thank the reviewer for checking this. We have revised the analysis and fixed the discounting rates for cost and effect, both at 3.5%, according to NICE guidance in the UK.

2.11. ***Line 280: I’m unclear - when the time-horizon is varied from 2 to 5 to 10 years, are the just varying the timeframe over which benefits and costs are counted, or are these also extending the period of time over which an individual is assumed to received LAAB-PrEP?***

**Author response:** We thank the reviewer for asking for this clarification. When the time-horizon is varied from 2 to 5 to 10 years, we vary the timeframe over which benefits and costs are counted, and at the same time we are also extending the period of time over which an individual is assumed to receive repeated doses of LAAB-PrEP, to cover the entire time horizon or their lifetime, whichever is the shorter. We thus refer to our simulation framework as ‘repeated dosing and repeated challenge’, as described in lines 173-184.

2.12. ***I wasn’t clear, does the model include ageing? And if so, does underlying mortality and COVID-19 mortality risk vary with age, which might be important particularly in the 10-year time-horizon analysis.***

**Author response:** We thank the reviewer for requesting this clarification. The model does not include ageing, but it does have two separate mortality rates (in the absence of COVID-19 and due to COVID-19) as inputs, as shown in Fig 4. The targeted vulnerable population features a higher mortality rate in the absence of COVID-19, which is less correlated with age.

2.13. ***Line 355: The authors write that “Typical cost-effectiveness analyses rely on well-defined input values that are used to determine whether a new intervention is cost effective. Our approach differs from this in that it includes uncertain input parameters yet can still be used to determine the features that have the greatest influence on the intervention’s potential health impact.” I think that the analysis presented is essentially a cost-effective analysis, and the lack of empirical data to inform input parameters is also common to such analysis.***

**Author response:** We thank the reviewer for this suggestion, and we have revised the text as suggested.

2.14. ***Line 367: “We found that shielding behaviour is highly influential in terms of whether LAAB-PrEP is cost-effective” From this it appears that the authors are making conclusions about cost-effectiveness. I would further add that I don’t think this conclusion is supported by the results - on the contrary from Fig 3 it appears that LAAB-PrEP would not be cost-effective in England using typical NICE thresholds under any of the scenarios presented.***

**Author response:** We thank the reviewer for pointing this out. We have redefined the low fatigue high replacement scenario by selecting new ranges for the parameter values such that the probability of cost-effectiveness is 90% at a WTP threshold of 30,000 GBP. Please see the revised Fig 3.

2.15. ***Line 375/376: As noted in point 2, I don’t think the current guidance represents a strict continuation of the shield guidance that was in place during the pandemic.***

**Author response:** We thank the reviewer for this point. We have revised the manuscript as suggested. Please also see our responses to 2.2.

2.16. ***Line 382: Costs of furlough and SSP are not costs that are born by the NHS, so I do not think they should be included in the base case analysis from a healthcare payer perspective but could be included in a sensitivity analysis using a societal perspective.***

**Author response:** We thank the reviewer for this suggestion, and we have revised the text as suggested. Please see the updated Fig 1.

2.17. ***Line 401. “However, this does mean that there will be scenarios in which even LAABs with intermediate effectiveness could be cost effective.” Again, I’m not sure this is supported by the results, as none of the scenarios presented appear likely to be cost-effective.***

**Author response:** In the updated Fig 3a, we found in the low fatigue and high replacement scenario, the probability of cost effectiveness for the LAAB-PrEP is 90%. Furthermore, we also explored the two-way sensitivity analysis for the trade-off between cost and efficacy of the LAAB-PrEP under the three scenarios defined by Fig 3a, because of the highly influential effect of population behaviour parameters on the cost effectiveness shown in Fig S5. We found that the plane of probability of cost effectiveness at the given efficacy and cost varied across the three scenarios. There will be settings in which even LAABs with intermediate effectiveness could be cost effective under the high fatigue scenario and the low fatigue high replacement scenario but not the low fatigue and low replacement scenario.

2.18. ***I think the discussion could include a fuller discussion of the study limitations. As one example, it seems very likely that “shielding” behaviour of immunocompromised individuals could provide protection against a wide range of pathogens beyond SARS-CoV-2, which as far as I can see are not considered in this analysis.***

**Author response:** We thank the reviewer for their suggestion. We have now added a discussion of the study’s limitations in lines 394-414.

2.19. ***Line 443/495: I’m not sure what is meant by the term “model-calibrated”. To me this suggests that the model has been in some way fitted to data, but I don’t think this is the case. Please could the authors clarify.***

**Author response:** We thank the reviewer for pointing out this confusion. We have revised the text to state ‘model-estimated’ in line 538 to distinguish the results from model-fitting.

2.20. ***Line 414: “...we have shown that the expected health and economic impact of LAAB PrEP for COVID-19 in immunocompromised patients will be driven, in order of decreasing importance...” I don’t think the analysis has separately shown that the health and economic impact are both influenced in this order.***

**Author response:** We thank the reviewer for pointing out this confusion. We have now separately described the influential parameters for the health and economic impact of LAAB-PrEP and revised the text in lines 454-465.

2.21. ***Line 512: As noted earlier, I could not see where the discounting sensitivity analysis is presented. Also, the reference for the values chosen is the NICE guidelines, but these do not appear to be correct. NICE recommends the use of 3.5% for both costs and QALYs, with 1.5% acceptable in some circumstances that are unlikely to apply to LAAB-PrEP (see page 82 of the guidance document.)***

**Author response:** Please also see our responses in 1.9.

2.22. ***Line 520: Here it says for the drug price a gamma distribution was used, but in the appendix, it says a uniform distribution was used. As noted earlier, I think this should not be treated as a stochastic parameter, but rather varied in scenario analysis.***

**Author response:** We thank the reviewer for pointing this out. That was a typo and has now been corrected. Also, we conducted the two-way sensitivity analysis as suggested. Finally, please also see our responses in 2.5.

**References**

1. Di Gessa, G Price, D. The impact of shielding during the COVID-19 pandemic on mental health: evidence from the English Longitudinal Study of Ageing. *Br J Psych* 2022;221:637-643.
2. Bachtiger, P et al. Determinants of shielding behavior during the COVID-19 pandemic and associations with well-being among National Health Service Patients: longitudinal observational study. *JMIR Public Health Surveill* 2021;7: e30460.
3. National Institute for Health and Care Excellence. NICE health technology evaluations: the manual 2022. Available from: <https://www.nice.org.uk/process/pmg36/resources/nice-health-technology-evaluations-the-manual-pdf-72286779244741>.
4. Hellmund C. The NICE HST process (1/3) – origins and current state of affairs. 2019. Available from: <https://source-he.com/the-nice-hst-process-1-3-origins-and-current-state-of-affairs/>.
