# Supplementary material for: Features influencing the health and economic impact of preventing COVID-19 in immunocompromised individuals
Source: PLoS Comput Biol. 2025 Jun 2;21(6):e1013072. doi: 10.1371/journal.pcbi.1013072 (PMC12129335; doi:10.1371/journal.pcbi.1013072)
Supplement: Attachment — Submitted filename: Reply to reviewers_R2_03032025.docx [file pcbi.1013072.s006.docx]

**Reply to reviewers’ comments**, **submission PLOS Computational Biology (PCOMPBIOL-D-24-00735R1)** "Features influencing the health and economic impact of long-acting antibody prophylaxis against COVID-19"

Reviewer #1:

***The authors have addressed my comments.***

**Author response**: We thank the reviewer for accepting our revisions.

Reviewer #2:

***2.1. Thank you for the opportunity to review this updated manuscript. I have some further questions and comments.***

**Author response:** We thank the reviewer for reviewing our latest revisions and are happy to address their further questions and comments.

***2.2. In response to my comment that the results presented did not appear to support the likelihood of LAAB-PrEP being cost-effective in the UK, the authors updated their definition of the “low-fatigue high-replacement” scenario so that his now appears to be cost-effective at £20,000 to £30,000 per QALY. I am somewhat uncomfortable with the scenario definition being amended to achieve a particular result. I think as a minimum it should be made clear in the methods that (some of) the scenarios have been selected in a purposeful way (i.e. to maximise the likelihood of a cost-effective outcome.)***

**Author response:** We thank the reviewer for their suggestion, and we have now made it clear in the Methods section that the “scenarios have been selected in a purposeful way (i.e. to maximise the likelihood of a cost-effective outcome.)”

***2.3. Given the above, I wonder if the authors might consider presenting the main results in a more theoretical way e.g. by focusing on the multi-way scenario analysis of the key unknown parameters, to highlight how the interaction of these parameters influences potential cost-effectiveness (similar to the new S5 figure.)***

**Author response:** We thank the reviewer for their suggestion and have moved Fig S5 into the main text as Fig 4 (all figures in both the main text and the supplementary information have been re-ordered). We also added the insights that the reviewer described in #2.2 to help the reader in understanding our work. Furthermore, we have adjusted the title of our manuscript to reflect the more theoretical approach taken.

***2.4. Related to the above, for the PSA, I am not sure how useful it is to incorporate extremely wide uncertainty e.g. uniform [1/50, 1/5] for death risk, or uniform [0.1,0.9] for LAAB-PrEP into a single simulation. If many parameters are varied over a very wide range that is not informed by empirical data, then the proportion of samples that are cost-effective tell us little about the probability that the intervention will be cost-effective.***

**Author response:** Immunocompromised patients represent a diverse population whose mortality risk varies widely, while the efficacy of LAAB-PrEP varies according to the viral strains in circulation. We deliberately varied most of the parameters of the model over a very wide range and focused our work on the sensitivity analyses. In this way, we were able to explore which parameter(s) have a dominant effect on cost effectiveness and how these parameters interact with each other to influence the cost effectiveness.

We also presented a case study, as shown in Fig 3d, where the parameters for mortality risk and many other parameters specific to each subgroup of the immunocompromised population are much narrower.

***2.5. I was interested to see in figure S5, that the effectiveness of LAAB-PrEP has only a small impact on whether it’s use is cost-effective. This is presumably because the majority of the benefit is down to behaviour change (reduced disutility of shielding) and the direct protective effect against Covid-19 does not actually matter much. In extremis it would seem that much of the benefit could theoretically be achieve by placebo at no cost. In practice presumably individuals would not stop shielding without having good evidence of at least some effectiveness from LAAB-PrEP. Is there any empirical evidence on which the link between risk reduction and shielding behaviour that could be used to inform this link?***

**Author response**: We thank the reviewer for these insights and have made the following changes to the Results section (lines 269–276) to aid readers’ understanding (shown below in red font). We have also made some adjustments to the Abstract and the Author Summary to reflect these changes in the main text.

“This highlighted the strength of the disutility of shielding and thus the strength of behaviour change in influencing the cost effectiveness of LAAB PrEP. We found that the effectiveness of LAAB-PrEP would only have a small impact on whether its use is cost effective. This is because the majority of the benefit would be due to behaviour change (reduced shielding), with the direct protective effect against COVID-19 contributing less.”
