# Supplementary material for: Features influencing the health and economic impact of preventing COVID-19 in immunocompromised individuals
Source: PLoS Comput Biol. 2025 Jun 2;21(6):e1013072. doi: 10.1371/journal.pcbi.1013072 (PMC12129335; doi:10.1371/journal.pcbi.1013072)
Supplement: Attachment — Submitted filename: Reply to reviewers_R3_04132025.docx [file pcbi.1013072.s007.docx]

**Reply to reviewers’ comments**, **submission PLOS Computational Biology (PCOMPBIOL-D-24-00735R2): Features influencing the health and economic impact of preventing COVID-19 in immunocompromised individuals**

Reviewer #2: ***The authors have addressed the majority of my comments.***

***There is one of my comments from the last review that the authors have not addressed or commented on. In the previous review I wrote that "The authors mention the NICE highly specialised technologies programme as a potential justification for using a £100,000 per QALY cost-effectiveness threshold. However, this programme is limited to very rare conditions with prevalence below 1 in 50,000 people and an eligible population of 300 – 500 people. From the INFORM study that has been used to guide the authors modelling assumptions, there are roughly 2 million people in England who met the immunocompromised definition. I continue to believe that this threshold does not provide a helpful indication of the potential cost-effectiveness of the proposed application of LAAB-PrEP in the England."***

***The authors have not addressed this in their response. In my opinion the best option would be to remove this threshold from the figures because for the general reader I think it creates a misleading impression of the likelihood of cost-effectiveness. However, if the authors wish the retain the use of this threshold then I suggest that my point above about the very small population that could potentially be eligible under this scheme is made explicit in both the main text and the figure captions where the threshold is presented.***

**Author response**: We thank the reviewer for accepting our revisions for addressing the majority of previous comments. Now we followed the reviewer’s suggestion and removed the 100,000 GBP threshold from all relevant figures throughout the manuscript to avoid any misleading for general readers.
